# Disparities in Incidence of Human Immunodeficiency Virus Infection Among Black and White Women — United States, 2010–2016

**DOI:** 10.15585/mmwr.mm6818a3

**Published:** 2019-05-10

**Authors:** Erin L.P. Bradley, Austin M. Williams, Shana Green, Ashley C. Lima, Angelica Geter, Harrell W. Chesson, Donna Hubbard McCree

**Affiliations:** ^1^Divsion of HIV/AIDS Prevention, National Center for HIV/AIDS, STD and TB Prevention CDC; ^2^Oak Ridge Institute for Science and Education, Oak Ridge, Tennessee; ^3^Division of STD Prevention, National Center for HIV/AIDS, STD and TB Prevention, CDC.

Incident human immunodeficiency virus (HIV) infections among adolescent females and women declined during 2010–2016, with the largest decrease (21%) occurring among black women (*1*). However, in 2016, although black women accounted for 13% of the U.S. female population, 60% of new HIV infections among women were in black women, indicating persisting disparities (*1*). CDC used the population attributable proportion (PAP) disparity measure to describe the proportional decrease in HIV infection among black and white women combined that would be realized if the group with the higher rate (blacks) had the same rate as did the group with the lower rate (whites) (*2*). Analyses indicated that an estimated 3,900 of 4,200 (93%) incident HIV infections among black women in 2016 would not have occurred if rates were the same for black and white women. The PAP disparity measure decreased from 0.75 in 2010 to 0.70 in 2016, suggesting that if incidence rates for black women were the same as those for white women, the annual number of incident HIV infections among black and white women would have been 75% lower in 2010 and 70% lower in 2016. Continued efforts are needed to identify and address social and structural determinants associated with HIV-related disparities to eliminate these disparities and decrease HIV incidence among black women.

CDC calculated the PAP disparity measure to assess trends in HIV infection disparities among black and white women in the United States from 2010 to 2016. HIV incidence and prevalence estimates for women and adolescent females aged ≥13 years from an HIV Supplemental Surveillance Report (*1*) were used to compare estimated incidence with the incidence had there been no racial disparity between blacks and whites (black-white disparity). The PAP disparity measure was calculated as the number of excess incident infections among black females divided by the total number of estimated incident infections among black and white females combined. Excess incident infections were determined as the estimated number of incident infections among black females minus the hypothetical number of incident infections (infections among black females in the absence of a black-white rate disparity). The hypothetical number of incident infections was obtained by dividing the HIV incidence rate in white females by 100,000 and then multiplying by the number of HIV-negative black females. To increase precision in the analyses because incident infection counts in the surveillance report were rounded to the nearest hundred, the estimated number of incident HIV infections was derived by dividing the surveillance report rate by 100,000, then multiplying by the number of females aged ≥13 years. Rates of HIV infection were defined as the estimated number of incident infections divided by the number of HIV-negative females aged ≥13 years, then multiplied by 100,000. This calculation was carried out for each year from 2010 to 2016. To assess changes in the PAP disparity measure between the beginning and the end of the study period, a z-statistic was calculated to test for statistically significant differences between the 2010 and 2016 measures. The z-statistic was calculated as the average difference between the 2016 and 2010 PAP disparity measures in the simulated data divided by the standard error of those differences. Simulations consisted of 10,000 calculations of the annual PAP measures, each using a random draw of the HIV incidence rate from a normal distribution (approximated using the relative standard errors from the surveillance report) (*3*).

From 2010 to 2016, the estimated incidence of HIV infection among black women and adolescent females decreased from 32.5 per 100,000 persons to 24.4; the rate among white women and adolescent females did not differ in 2016 (1.6) compared with that in 2010 (1.6) and ranged from 1.4–1.7 during that time. The PAP disparity measure decreased from 0.75 in 2010 to 0.70 in 2016. This change suggests that if incidence rates for black women were the same as were those for white women, the annual number of incident cases of HIV infection among black and white women would have been 75% lower in 2010 and 70% lower in 2016 (Table). The 7% decrease in the PAP disparity measure from 2010 to 2016 (p = 0.15) indicates that the percentage of incident HIV infections attributable to racial disparities between black and white women decreased by about 7% over this period. Thus, in 2016, an estimated 3,900 of 4,200 (93%) incident HIV infections among black women would not have occurred if rates were the same for black and white women.

**TABLE:** Population attributable proportion (PAP) for human immunodeficiency virus (HIV) incidence among black and white women and adolescent females aged ≥13 years, by race — United States, 2010–2016

Year	No. of incident HIV infections* (rate^†^)		Excess infections among blacks	PAP^§^	% Change 2010 to 2016^¶^	P-value
	Blacks	Whites				
2010	5,300 (32.5)	1,400 (1.6)	5,000	0.75	−7	0.15
2011	5,000 (30.7)	1,300 (1.5)	4,800	0.75		
2012	4,700 (28.6)	1,300 (1.5)	4,500	0.74		
2013	4,400 (26.0)	1,200 (1.4)	4,100	0.74		
2014	4,000 (23.4)	1,300 (1.5)	3,700	0.70		
2015	4,100 (23.7)	1,500 (1.7)	3,800	0.68		
2016	4,200 (24.4)	1,400 (1.6)	3,900	0.70		

* Number of incident infections from an HIV Surveillance Report (https://www.cdc.gov/hiv/library/reports/hiv-surveillance.html). Incident infection counts rounded to the nearest hundred.

^†^ Infections per 100,000 population. To increase precision in the analyses, rates were calculated as the estimated number of incident HIV infections not rounded to the nearest hundred (surveillance report rate divided by 100,000, multiplied by the number of females aged ≥13 years) divided by the number of HIV-negative females aged ≥13 years, then multiplied by 100,000.

^§^ The PAP disparity measure reflects the percentage of HIV infections attributable to racial disparities in HIV incidence between black and white women and adolescent females aged ≥13 years. The PAP measure was calculated as the number of excess incident infections among black females divided by the total number of estimated incident infections among black and white females. Excess incident infections among black females refers to the estimated number of incident infections among black women minus the hypothetical number of incident infections that would have occurred among black women if their HIV incidence rate were the same as that of white women. The hypothetical number of incident infections in the absence of a black-white disparity in rates was calculated by dividing the HIV incidence rate in white females by 100,000 and multiplying by the HIV-negative black female population.

^¶^ The percent change from 2010 to 2016 was calculated as the difference between the 2016 and 2010 PAP values, divided by the 2010 PAP value.

## Discussion

The declines in incidence of HIV infection among black women and adolescent females signal some progress toward reducing racial disparities among women, and these findings are consistent with previous research that indicated reductions in racial/ethnic disparities in diagnosis of HIV infection among women during 2010–2014 using different measures of disparity (absolute rate difference, diagnosis disparity ratio, and index of disparity) (*4*). However, notable black-white disparities among women persist. In 2016, an estimated 93% of incident HIV infections among black women would not have occurred if the incidence rate for black women were as low as the rate for white women. Estimates of the annual PAP disparity measure during 2010–2016 suggest that eliminating black-white disparities in incident HIV infections among women and adolescent females would have achieved a decrease in overall incidence among black and white women of 75% in 2010 and 70% in 2016. This finding highlights the contribution of racial/ethnic disparities to overall HIV infection rates among women and adolescent females and underscores the importance of further reducing, or eliminating, these differences.

Reducing and monitoring HIV-related disparities are important national goals (*5*). Tailored strategies to reduce disparities in incidence among women should address social and structural determinants, including inequitable access to health care, HIV-related stigma, and comparatively high background prevalence of certain sexually transmitted infections (*6*,*7*), that increase the risk for HIV infection among black women. Because most HIV infections among black women occur through heterosexual transmission (*1*), strategies that also effectively engage heterosexual and bisexual men are important. Social and structural determinants create or sustain disparities in HIV infection, treatment, and care. For example, compared with their white counterparts, black women and men experience longer delays in diagnosis (*8*) and are less likely to be virally suppressed (i.e., <200 copies of viral RNA per mL of blood) (*9*,*10*). Targeted measures that address reducing transmission through viral suppression and preventing acquisition through biomedical and behavioral interventions (e.g., preexposure prophylaxis [PrEP] and condom use; and providing adequate treatment once HIV infection is diagnosed) will play important roles in reducing disparities.

The findings in this report are subject to at least five limitations. First, estimates of HIV incidence are subject to model assumptions and data completeness (*1*). Second, only one measure of disparity was used, limiting a more comprehensive analysis of racial/ethnic disparities in incidence of HIV infection among women and adolescent females. Using other measures of disparity could provide alternative results. Third, the p-value calculated for the 7% change in the PAP might be overestimated because it assumed no correlation in the error of estimated incidence within racial groups over time. This implies that the error in estimating the 2010 incidence among black women is unrelated to the error in estimating the 2016 incidence among black women. Fourth, although the PAP disparity measure has a straightforward interpretation and quantifies excess HIV infections among black females, this study does not yield additional insight into what structural or policy changes are needed to eliminate disparities. Finally, incidence in only two racial groups was compared, whereas disparities might exist among other racial/ethnic groups.

Despite these limitations, findings from the PAP disparity measure analyses enhance the measurement of HIV disparities among women and adolescent females by quantifying the number of incident HIV infections that might have been prevented in the absence of racial disparities. This information lends support for strengthening HIV prevention and care efforts for heterosexual black females and males to continue progress toward closing the gap in racial disparities in HIV infection among women. Such gains are needed to achieve the U.S. Department of Health and Human Services’ goal of ending the HIV epidemic in the United States by 2030* and prevent deaths related to acquired immunodeficiency syndrome.

**Summary**

**What is already known about this topic?**

Rates of human immunodeficiency virus (HIV) infection among all women have declined since 2010, but rates among black women remain higher than do those among white women.

**What is added by this report?**

A population attributable proportion analysis found that in 2016, an estimated 3,900 of 4,200 (93%) incident HIV infections among black women would not have occurred if the incidence for black women were the same as that for white women.

**What are the implication for public health practice?**

Reducing racial disparities among women is needed to achieve broader HIV control goals. Addressing social and structural determinants of health and applying tailored strategies to reduce HIV incidence in black women and their partners are important elements to achieving health equity.

## Footnotes

* https://www.hiv.gov/federal-response/ending-the-hiv-epidemic/overview.
